# Open-label placebos reduce weight in obesity: a randomized controlled trial

**DOI:** 10.1038/s41598-024-69866-7

**Published:** 2024-09-12

**Authors:** Michael Schaefer, Anja Kühnel, Sören Enge

**Affiliations:** MSB Medical School Berlin, Rüdesheimer Str. 50, 14197 Berlin, Germany

**Keywords:** Obesity, Overweight, Placebo, Open-label placebo, Eating behavior, Psychology, Human behaviour, Weight management

## Abstract

Obesity is a major public health problem worldwide. Different approaches are known to face this problem, for example, dieting, surgery, or drug interventions. It has also been shown that placebos may help to reduce weight and hunger feelings, but the use of placebos is linked to problems with respect to the patient-healthcare-provider relationship. However, recent studies demonstrated that even placebos without deception (open-label placebos) affect symptoms such as pain, anxiety, or emotional distress. Here we aimed to examine whether an open-label placebo may help to lose weight in obesity. Our study included fifty-seven overweight and obese patients who aimed to lose weight using a combination of diet and sports. Patients were randomly divided into two groups. Participants in the open-label placebo group received two placebos each day. A treatment-as-usual group received no pills. Primary outcome included changes of body weight. Secondary outcomes were change of eating behavior and self-management abilities. After 4 weeks we found that participants in the open-label placebo condition lost more weight than the treatment-as-usual group. Furthermore, OLP treatment affected eating behavior. No effects for self-management abilities were found. Although further research is necessary, open-label placebos might help individuals to lose weight.

## Introduction

Obesity has become one of the leading public health problems in the world, with still rising rates^1,2^. It also represents an economic burden, as obese individuals have 30% higher medical costs than individuals with normal weight^3–7^. Different strategies are discussed to help people losing weight, including dieting, drug interventions, psychological approaches, or surgery^8–11^. Recent research also demonstrated that placebo effects may play an important role in obesity treatments. For example, a recent systematic review reported that patients in sham bariatric surgery exhibit 71% of the weight loss that active surgery groups showed, pointing to strong nonspecific or placebo effects^12^. In addition, Panayotov reported placebo effects for obesity in an imaginary low-calorie diet^13^ (see^14^ for a methodological commentary on this paper). Furthermore, placebo effects for feelings of hunger have been shown. For example, in a study by Crum et al. participants were asked to consume a 380-cal milk shake with a label of pretending either 140 cal (the “sensible shake”) or 620 cal (the “indulgent shake”)^15^. Consuming the “indulgent shake” led participants to feel greater satiety (self-reported and measured with ghrelin). The authors conclude that mindsets, not nutrients, affected the ghrelin response in their study. The author’s hypothesis that mindsets can be crucial for our eating behavior and health is also supported by another intriguing study by this group. Crum and Langer measured physiological health variables of 84 female room attendants working in hotels. Half of them were then informed that the work they do is “good exercise and satisfies the Surgeon General’s recommendations for an active lifestyle”. The other half did not receive this information. Four weeks after this intervention the informed group showed a decrease in weight, blood pressure, body fat, waist-to-hip ratio, and BMI. This is remarkable because the actual behavior or food intake of the subjects did not change. The authors conclude that the impact of exercise on our health may be grounded at least in part by the placebo effect^16^. Chang et al. provided further evidence that placebo effects affect our health and eating behavior. They gave one group a purported weight-loss supplement, whereas the control group received a placebo. In fact, both groups received a placebo pill. The authors found that taking the allegedly weight-loss supplements made participants eat more and prefer larger quantities of sugar in their drinks than the controls. Thus, the weight-loss supplement placebo decreased dietary self-regulation^17^. Similar results have been reported in a recent study by Hoffmann et al.^18^ and also in an imaging study by Khalid et al.^19^.

However, using placebos in a clinical context is related to ethical problems, because physicians do not inform their patients that they receive placebos^20,21^. This is problematic, considering the importance of the patient–healthcare provider relationship or the general role of the patient as it is understood today (violation of the principles of informed consent). The view that deception is essential for seeing placebo responses has now been challenged by research in the last decade. Several studies reported that placebos given without deception (open-label placebos, OLPs) might also have beneficial effects, e.g., for (chronic) pain, emotional distress, and anxiety^22^. Since the application of OLPs is not involved in ethical problems (even though conventional placebos may be preferred^23^), OLPs may offer important new treatment possibilities^24^. Although negative findings have also been reported (e.g., for nausea^25^ and wound healing^26^, but at least for the last conventionally placebo effects are also not known) meta-analyses in clinical samples report remarkable effect sizes^22,27^.

Do OLPs also help in obesity to lose weight and reduce hunger feelings when trying to lose weight? Given the complex relationships between psychological factors and appetite or food consumption we showed above for conventional placebos, this may seem reasonable. The present study ties in there. We conducted a randomized controlled trial to test whether an OLP treatment affected weight and hunger feelings in obese and overweight patients, who were trying to lose weight by reducing their caloric intake and following a sports strategy. Before and after the trial we measured weight and hunger feelings. Based on previous results we hypothesized that patients in the OLP group will lose more weight and will feel less hunger than a group without receiving OLPs.

## Materials and methods

### Participants

**Figure 1:**
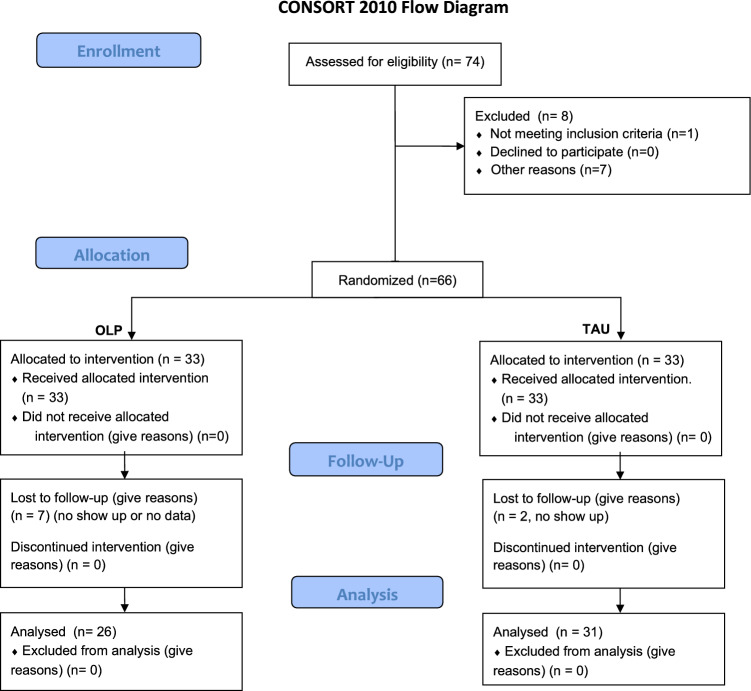
Flow diagram of enrollment.

Fifty-seven individuals with no neurological or psychiatric history participated in this trial (mean age 33.04, standard deviation ± 14.18 years, 24 females, see FlowChart with details about enrollment, Fig. 1). The study adhered to the Declaration of Helsinki and was approved by the ethics committee of the Medical School Berlin. All participants gave written informed consent. The study was pregistered at the German Clinical Trial Registration (DRKS00033046, 15.11.2023).

Inclusion criteria were age between 18 and 60 and a BMI of more than 25. Exclusion criteria were any known psychiatric or neurological history, eating disorders, and pregnancy. Participants were recruited via local universities and social media.

### Study design

The study consisted of a 4-week randomized controlled trial comparing an OLP with a “treatment-as-usual” group (TAU). The study was announced as a scientific study to examine psychophysiological interactions in patients, who want to lose weight through exercise and a reduced-calorie diet. When enrolled in the study all participants had to follow a concrete sports strategy for losing weight as well as the intention to change their eating habits and switch to a lower-calorie-diet (specifically reducing sweet and salty snacks and avoiding fast food) at the time when they start the study. Participants received detailed guidance on the exercise strategy and how to change their lifestyle (based on recommendations of the WHO). A combination of changing eating behavior and exercise is known as an evidence-based weight loss intervention (e.g.,^28^) As we researched the role of OLPs on weight loss this evidence-based strategy is fundamental. We measured weight and eating behavior as well as self-management abilities at the beginning and at the end of the trial.

Sample size was determined by calculations based on previous OLP research in patients^22^. An alpha error probability of 0.05, an effect size of 0.7, and a desired power of 0.80 resulted in a minimum number of 52 participants, according to a power calculation using G-power^29^.

### Procedure

Participants first completed baseline assessments including their BMI (weight and size) and questionnaires with respect to their eating behavior and self-management abilities (see below). Then all participants were briefed about the placebo effect identical to previous research^30^. Thus, we emphasized that placebos are inactive substances and that they contain no medications, but placebo effects may still be powerful, that the body may automatically respond to taking placebo pills, that a positive attitude is helpful (but is not necessary), and that placebo pills need to be taken faithfully. Furthermore, we also provided information that previous research has demonstrated that even placebos without deception resulted in positive effects. This procedure is identical to most of previous OLP studies (e.g., ^30^). Participants finally watched a news video that described scientific studies about OLP effects (analogue to^31^). Thus, although we were transparent by informing participants that the placebos were inert substances, we did not guide the expectations of the patients with respect to our specific study aims (e.g., reduce hunger feelings) or operationalization of assessments, but rather informed our patients about the nonspecific therapeutic benefits of the OLP treatment (similar to previous research, e.g.,^32^). Then we randomized the participants into two groups, an OLP and a TAU group. Participants in the OLP group received 56 placebo pills with the instruction to take one pill in the morning and one for the night. Placebo pills came in a white box labeled with the logo of the local university and consisted of cellulose microcrystalline in a vegetable capsule. Participants in the TAU group received no treatment. Furthermore, the importance of the control condition for the research goals was explained to its participants. Patient-healthcare provider relationship and amount of contact time was kept comparable for all groups.

### Outcome measures

Primary outcome measure was the BMI of the participants, which was calculated by measuring the weight at the first (before starting the trial) and at the second measurement. Secondary outcome measure was eating behavior. Eating behavior was examined by using the FEV questionnaire^33^, which is a German version of the three-factor eating questionnaire^34^. The FEV consists out of three scales: cognitive restraint of eating, disinhibition, and feelings of hunger. The first two scales have been shown to predict successful weight loss. It is an established questionnaire with good psychometric properties^33,34^. A further secondary outcome measure was the FERUS^35^, which provides a global score for self-management skills (coping, self-efficacy, introspection, hope, and self-verbalization) and two further scales, resource change motivation and perceived social support (five-point scale).

In addition, we examined the belief in placebos exploratory to test whether this variable is linked to OLP effects. We used four items to assess the general belief in placebos, which have been used in previous research on OLPs (identical to^36^, six-point-scale). Finally, participants of the TAU group were asked whether they were disappointed to have been randomized to the control group.

### Randomization and blinding

Randomization of group assignment was performed according to a computer-generated random number sequence. While the participants obviously knew whether they were in the OLP or TAU group, the experimenters involved in the further processing of the study were blinded to the group assignment.

### Statistical analysis

Hypotheses were tested by calculating change scores from pre to post (separately for weight, FEV, and FERUS) and by using baseline scores as a covariate (ANCOVA). Furthermore, we tested whether groups were different at baseline by using unpaired t-tests.

## Results

Table 1 depicts demographic details and baseline characteristics of the participants. Statistical testing (t-tests) for baseline data revealed no significant differences of BMI, eating behavior, self-management abilities and other measures between the two groups (*p* > 0.10) at baseline. All participants confirmed that they have followed the diet and exercises strategy.

**Table 1:** Demographics and baseline characteristics.

Characteristic	OLP	TAU
N	26	31
BMI (at baseline)	28.29 ± 2.68	29.81 ± 5.80
Belief in Placebo	4.22 ± 1.13	4.42 ± 0.98
Smoking	5	8
FEV-cognitive constraints (baseline)	9.77 ± 2.53	9.74 ± 2.85
FEV-disinhibition (baseline)	5.42 ± 2.21	6.16 ± 2.78
FEV-hunger (baseline)	5.54 ± 2.10	5.97 ±3.02

Mean ± standard deviations.

### Primary outcomes: BMI changes

**Figure 2:**
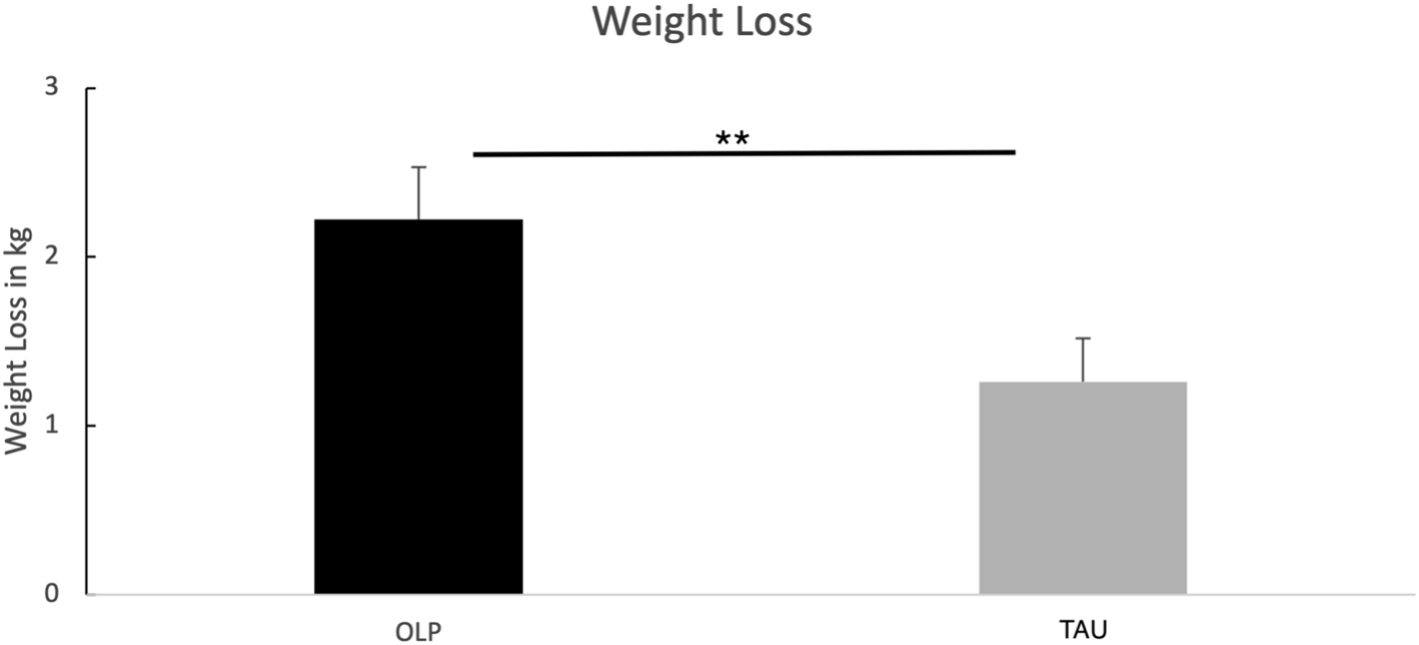
Outcome of weight changes after 4 weeks (means and standard errors). Results demonstrate higher weight loss for the OLP group.

Analyzing weight of the patients from pre to post showed that both groups successfully lost weight after 4 weeks. Results of an ANCOVA demonstrates that the OLP group lost significantly more weight than patients in the TAU group (change score of weight reduction pre to post, OLP: 2.22 ± 1.59 kg, TAU: 1.26 ± 1.43 kg; ANCOVA with baseline scores as covariates: F(1,54) = 6.91, *p* = 0.011, partial eta^2^ = 0.11, see Fig. 2). An ANOVA with repeated measurements (factors time and group) revealed comparable results (interaction between time and group, F(1,55) = 5.64, *p* = 0.021, partial eta^2^ = 0.09).

### Secondary outcomes: eating behavior and self-management abilities

**Figure 3:**
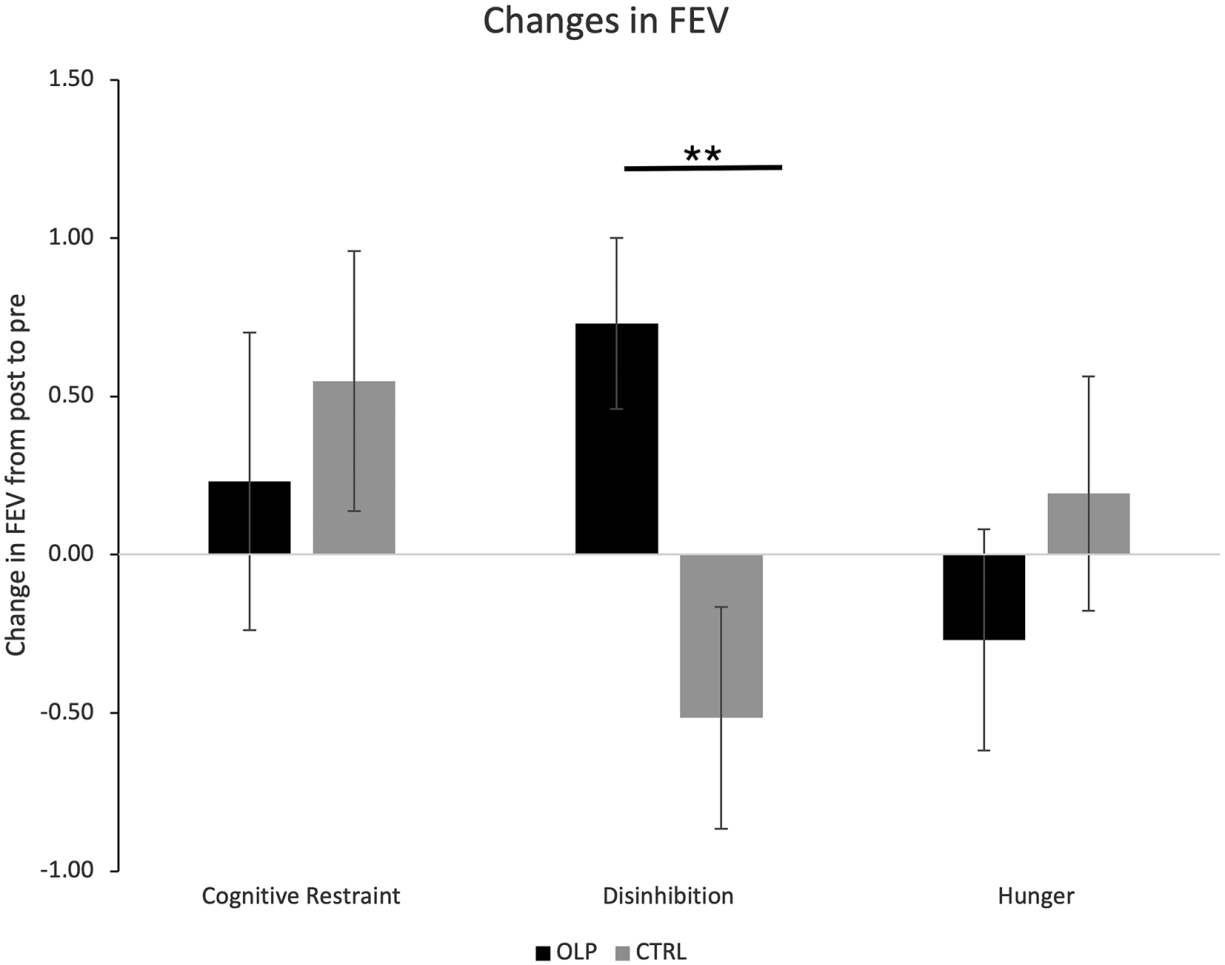
Changes in outcome parameter eating behavior (measured with FEV questionnaire). Results show increased control of eating behavior and less disinhibition of eating, accompanied with increased hunger feelings for the TAU group, as expected and well-known for patients during a diet. However, in the OLP group this pattern was different. Disinhibition was not lower and hunger feelings (no statistical difference) did not increase. Thus, the OLP treatment seems to result in different eating behavior compared with patients of the TAU group.

Analyzing eating behavior (FEV questionnaire) showed the expected and well-known pattern of changes in the TAU group. Thus, patients in this group showed increased cognitive control and reduced disinhibition of eating behavior after 4 weeks, which was accompanied by an increase of hunger feelings. Results for the OLP group showed a different picture (see Fig. 3). Here the patients also showed higher cognitive control, but in contrast to the TAU group even higher disinhibition and less hunger feelings than the patients of the TAU group (only significant for disinhibition).

Thus, after 4 weeks cognitive control of eating behavior was higher for both groups (no group effects, *p* > 0.10), whereas disinhibition of eating behavior decreased in the TAU group and increased in the OLP group (OLP: 0.73 ± 1.37, TAU: − 0.52 ± 1.93; ANCOVA: F(1,54) = 6.19, *p* = 0.016, partial eta^2^ = 0.09; similar results for ANOVA with repeated measurements). Furthermore, the TAU group experienced more hunger feelings after 4 weeks, whereas the OLP group reported even less hunger feelings than before. However, this difference failed to reach the level of significance (change scores OLP: − 0.27 ± 1.67, TAU: 0.19 ± 2.06; ANCOVA: *p* > 0.10, partial eta^2^ = 0.02; similar results for ANOVA with repeated measurements). None of these changes in eating behavior were correlated with the amount of weight loss over the 4 weeks.

OLP treatment did not affect self-management abilities (FERUS, all *p*s > 0.10). Thus, self-management abilities increased for both groups in a similar way (see Table 2). No significant correlations of the FERUS measures with weight lost were found.

**Table 2:** Outcome at 4 weeks endpoint.

	OLP	TAU
Weight loss (in kg)	**2.22 ± 1.59**	**1.26 ± 1.43**
Cognitive constraints (FEV, post–pre)	0.23 ± 2.39	0.55 ± 2.31
Disinhibition (FEV, post–pre)	**0.73 ± 1.37**	− **0.52 ± 1.93**
Hunger (FEV, post–pre)	− 0.27 ± 1.76	0.19 ± 2.06
Self-management skills (FERUS, post–pre)	5.02 ± 13.76	4.72 ± 17.89
Perceived social support (FERUS, post–pre)	− 1.15 ± 3.73	0.19 ± 4.46
Resource change motivation (FERUS, post–pre)	0.93 ± 4.02	1.14 ± 4.48

Mean ± standard deviations of change scores, significant differences in bold.

Last, we controlled whether any of our measures (weight, FEV) were linked to the belief in placebos. Calculating group specific Pearson correlations revealed no significant relationships (all *p*s > 0.10).

## Discussion

This study aimed to test whether an OLP treatment is successful to reduce weight in obesity. We found that both OLP and TAU groups were successful in losing weight, but patients of the OLP group showed significantly higher weight loss. Moreover, eating behavior after weight loss was different in patients of the OLP group.

Previous studies showed that conventional placebo treatments affected weight loss or feelings of hunger^15–18,15,37^. Recent studies also showed effects of OLPs for different clinical symptoms^22^. The current study aimed to test whether participants with obesity may also benefit from OLPs. Our results demonstrated that participants in the OLP condition lost more weight than the TAU group. Therefore, participants with overweight or obesity seem to profit from an additional OLP treatment when trying to lose weight.

Our study also revealed effects with respect to eating behavior. When trying to lose weight, e.g., by following a low-calorie diet, patients usually show higher control and less irritability of their eating behavior, which is seen as a good predictor of losing weight^34^. Furthermore, dieting is often accompanied with hunger feelings^34^. Data of the FEV questionnaire demonstrates this well-known pattern for the TAU group. In contrast, patients of the OLP group revealed a different picture. In this condition cognitive control (or restraint) also seemed to increase, but disinhibition (or irritability) of eating behavior was significantly higher (instead of lower) at the end of the diet. Furthermore, the OLP group seems to suffer less hunger (but this difference failed to reach the level of significance). Thus, one could speculate that the participants of the placebo group may have felt less hunger, therefore it was easier for these patients to hold on to their strategy of losing weight, even without paying attention to inhibit their eating behavior.

Eating behavior of the OLP group was in particular different with respect to the dimension disinhibition, which was raised in patients of the OLP group (in contrast to the control patients). Interestingly, this dimension of eating behavior was not affected by OLPs in our previous pilot study (which used the identical study design but included participants with slight overweight)^38^. Similar to the current trial our previous study found that the OLP group felt less hunger and lost more weight (no statistical difference, which might be explained by the shorter time period of the trial or simply by the lower weight of the participants).

It seems remarkable that patients in the OLP group were even more disinhibited after 4 weeks of trying to lose weight. The scale disinhibition refers to a loss of control in food intake, pointing to habitual, emotional, or situational susceptibility to disinhibition^39^. For example, situational susceptibility refers to specific environmental cues such as social occasions, whereas items such as “When I’m sad, I often eat too much” describe an emotional dimension of disinhibition. We speculate that the reason for seeing an effect here, in contrast to our previous study, may point to altered eating behavior in obesity^40^. Numerous studies have demonstrated that eating behavior is different in individuals with obesity^41,42^. Altered eating behavior in our obesity sample can also be seen when comparing baseline scores of the FEV questionnaire with norms of healthy non-obesity participants^43^. The different physiology in obesity may also account for the smaller effects on hunger feelings compared with our previous study^41,42^. It has been demonstrated that obesity-induced inflammation in the hypothalamus causes cellular resistance to insulin and leptin, resulting in problems of the hypothalamus’ function to regulate the homeostatic appetite system^41,42^. Moreover, antibodies (immunoglobulin), which are needed to break down the hunger hormone ghrelin, may have different properties in obesity^44^. However, it seems remarkable that losing weight in the OLP group seems to take place even without improved controlling of disinhibiting events. This may be in line with studies on conventional placebos, which also report loss of weight predominantly due to an altered mindset rather than changing other factors (e.g.,^16^).

Possible effects of an OLP treatment on hunger feelings may be very interesting. Feelings of hunger when trying to lose weight are a frequent problem and may be explained by the hormone ghrelin, which is engaged in food intake and body weight regulation. Ghrelin is understood as an indicator for energy insufficiency. When the stomach is empty or energy intake is low, ghrelin is secreted in the stomach and then transported via the bloodstream to the brain, where it binds with arcuate nucleus and the hypothalamus to evoke the feeling of hunger^15^. If food is consumed and energy intake increases, the ghrelin levels will be suppressed to signal the brain to reduce appetite and food intake, associated with the sensation of satiety^45^. However, importantly ghrelin is also known to increase with weight loss and decrease with weight gain. Thus, ghrelin seems to change in a compensatory fashion to weight change, probably in order to regain diet induced weight loss^46,47^. Therefore, a result of many food restriction diets is that the weight loss is accompanied by an increase of ghrelin levels (e.g.,^48^), which may then result in problems to keep the reduced weight. OLPs might help obesity in a way that is different from other strategies, in particular with respect to hunger feelings. Thus, it seems promising to test in future studies whether an OLP treatment may help patients not only to lose weight but also to keep this weight over time.

Based on the present data it is difficult to speculate about possible mechanisms of an OLP treatment. Previous studies suggested that expectation, which is often suggested as an important mechanism for conventional placebos^49^, may not play a similar role for OLP effects^50,51^ (see also^52^ for a general discussion of mechanisms in placebo studies). The present study did not find any relationships of the effects with the general belief in placebos. In this context it may be noteworthy that the present study did not guide expectations by explicitly informing the participants about the concrete hypothesis, rather we informed them about possible non-specific effects of the placebo treatment. However, the exact mechanisms of OLPs remain to be cleared^53^.

The present study reports medium effect sizes (partial eta^2^ = 0.11) that are comparable to other OLP studies with clinical samples. For example, recent meta-analyses found OLP effects of d = 0.7 or d = 0.5, respectively^22,27^. However, further studies are necessary to reveal the mechanisms and underlying neural substrates of OLP effects, which is important to evaluate the potential implications of this novel treatment.

Several limitations of our study may apply. Our findings are based on a relatively small sample size and need to be replicated by studies including larger samples. Furthermore, a follow-up would be necessary. The question how long the effects will last is of particular interest in individuals trying to lose weight. In addition, further comparisons with conventional placebos would provide important information about the impact and effect size of OLP treatments in obesity. Another limitation is that data about the adherence to the strategy to lose weight by doing exercises and changing eating behavior were self-reported. Since participants in the current study were not in a hospital, it was difficult to ensure that all groups paid equal attention to compliance with the strategy. Finally, the current study provides only little information about the mechanisms of this OLP treatment. Future studies could include physiological dimensions (e.g., measurement of ghrelin or PYY) as well as further psychological dimensions (e.g., role of expectation and believe specifically in OLPs).

This is the first study testing OLPs in obesity. We found that the treatment resulted in greater weight loss compared with a TAU group. Given that OLPs are not linked to any side effects and can be administered in an ethically unproblematic way, we believe that the results are encouraging to further examine this treatment in obesity (see also^37,54^), a group of individuals that is still increasing in many parts of the world.

## Data Availability

The data presented in this study are available on request from the corresponding author.
